# The role of microbial interactions on rhizobial fitness

**DOI:** 10.3389/fpls.2023.1277262

**Published:** 2023-10-09

**Authors:** Margarita Granada Agudelo, Bryan Ruiz, Delphine Capela, Philippe Remigi

**Affiliations:** Laboratoire des Interactions Plantes Microbes Environnement (LIPME), Université de Toulouse, INRAE, CNRS, Castanet-Tolosan, France

**Keywords:** rhizobia, symbiosis, nitrogen fixation, microbial communities, microbe-microbe interactions, eco-evolutionary dynamics

## Abstract

Rhizobia are soil bacteria that can establish a nitrogen-fixing symbiosis with legume plants. As horizontally transmitted symbionts, the life cycle of rhizobia includes a free-living phase in the soil and a plant-associated symbiotic phase. Throughout this life cycle, rhizobia are exposed to a myriad of other microorganisms that interact with them, modulating their fitness and symbiotic performance. In this review, we describe the diversity of interactions between rhizobia and other microorganisms that can occur in the rhizosphere, during the initiation of nodulation, and within nodules. Some of these rhizobia-microbe interactions are indirect, and occur when the presence of some microbes modifies plant physiology in a way that feeds back on rhizobial fitness. We further describe how these interactions can impose significant selective pressures on rhizobia and modify their evolutionary trajectories. More extensive investigations on the eco-evolutionary dynamics of rhizobia in complex biotic environments will likely reveal fascinating new aspects of this well-studied symbiotic interaction and provide critical knowledge for future agronomical applications.

## 1 Introduction

Ecological systems are complex. They involve a multitude of organisms that can interact with each other. These interactions, ranging from antagonism to mutualism, strongly influence the fitness of each individual and, consequently, the structure of the communities in which they live. Microbial communities, termed microbiomes, in particular have received much attention because of the fundamental role they play in Earth’s biogeochemical cycles and in plant and animal health. Common mechanisms governing interactions within microbiomes include competition for resources, predation, the production of antagonistic/toxic molecules, cross-feeding processes, the production of public goods, or the formation of protection structures such as biofilms (Konopka, 2009; Pierce and Dutton, 2022). These interactions are particularly prevalent and significant in dense host-associated microbial communities, such as the mammalian gut or the rhizosphere (Hassani et al., 2018; Coyte and Rakoff-Nahoum, 2019; Kern et al., 2021; Chepsergon and Moleleki, 2023). In these ecosystems, positive or negative interactions between microbiome members can allow or inhibit, respectively, the proliferation of pathogens or beneficial microbes, with important effects on host health. For example, some rhizospheric bacteria were shown to inhibit the growth of fungal pathogens and protect plants against disease (Carrión et al., 2018; Durán et al., 2018).

Notable members of the rhizosphere community are rhizobia. These bacteria are able to form mutualistic associations with legume plants, during which they fix atmospheric nitrogen to the benefit of the host, in exchange for carbon compounds from photosynthesis (Poole et al., 2018). Rhizobia are gram-negative bacteria classified in 18 different genera of Alpha- and Beta-proteobacteria including *Rhizobium, Sinorhizobium, Bradyrhizobium, Mesorhizobium, Azorhizobium, Cupriavidus*, and *Paraburkholderia* (Masson-Boivin et al., 2009; Tang and Capela, 2020). Rhizobia are horizontally-transmitted symbionts. Their biphasic life cycle is composed of a free-living saprophytic phase, where rhizobia are part of the soil and rhizosphere microbiomes, and a symbiotic phase in their host. Soil bacteria are attracted to the germinating seeds or the mature roots following the perception of chemoattractants present in plant exudates (Compton and Scharf, 2021). In the plant, rhizobia are hosted in specific root organs, called nodules. Nodule formation is initiated by the exchange of compatible signals between rhizobia and legumes (Walker et al., 2020). In most rhizobia, the expression of *nod* genes, responsible for the synthesis of lipochito-oligosaccharides called Nod Factors (NF), is induced by specific flavonoids exuded by host plants. NF, whose structures vary between rhizobium strains, are then specifically recognised by plant receptors. The perception of NF allows the entry of bacteria in root tissues, where they start to proliferate extracellularly. NF perception and downstream signaling also trigger a plant development program, which leads to nodule organogenesis. In most legumes of the Papilionoideae and Mimosoid clades (Sprent, 2009; De Faria et al., 2022), rhizobia are then engulfed in the cytoplasm of nodule cells, where they form structures surrounded by the plant plasma membrane called symbiosomes. Rhizobia differentiate into bacteroids that fix nitrogen, and persist for several weeks or months within nodule cells. In legumes of the Inverted Repeat-Lacking Clade and Dalbergioid clade, this differentiation is terminal, meaning that bacteroids cannot resume growth after nodule senescence (Mergaert et al., 2006; Czernic et al., 2015; Montiel et al., 2016). During nodule senescence, undifferentiated bacteria and non-terminally differentiated bacteroids present in nodules are released and can recolonise the soil and the rhizosphere. The ability of a given rhizobial strain to successfully complete the different steps of this complex life cycle will determine how fit it is in its current environment. Studying the different factors governing rhizobial fitness is critical to understand the diversity, ecology and evolution of these important plant symbionts.

All along their life cycle, rhizobia interact with other microorganisms composing the soil, rhizosphere and nodule microbiomes, and are therefore involved in a diversity of interactions that affect their fitness either directly or indirectly through plant-mediated mechanisms. This review focuses on how the microbial community context, *i.e.* the ecological interactions between rhizobia and other microorganisms, contributes to determining rhizobial fitness. We first discuss the notion of fitness in the case of rhizobia and then describe how the diverse rhizobia-microorganisms (including rhizobia-rhizobia) interactions affect the fitness of rhizobia and their evolutionary dynamics.

## 2 The multiple facets of rhizobial fitness

Fitness is a central notion in evolutionary biology, as it measures how well a genotype performs in terms of survival and reproduction in a given environment. Yet, experimental measurements of fitness can be challenging. Even in seemingly simple systems (Vasi et al., 1994), bacterial fitness is dependent on several phenotypic traits (called ‘fitness components’) that will determine a genotype’s performance at the different steps of the life cycle (Orr, 2009). However, the life history traits that are measured and considered as best fitness proxies can differ between studies and experimenters. This is typically the case for rhizobia. The different measurable fitness components include (i) proliferation and survival in the soil and the rhizosphere, (ii) nodulation proficiency and competitiveness, (iii) proliferation and survival within nodules, and (iv) bacterial release from nodules during senescence. Below we highlight salient aspects of some of these different fitness measures.

Understandably, literature on rhizobial fitness has put a lot of emphasis on bacterial traits governing the association with host plants. First, the nodulation step is a major determinant of fitness for rhizobia, since it represents a strong selective bottleneck for rhizobial populations and rhizobia founding nodules will leave many more descendants than those staying in the rhizosphere (Denison and Kiers, 2011). Although nodules are usually considered to be founded by one single bacterium, mixed nodules, hosting several different rhizobial strains, can also be found in proportions that vary depending on the plant growth substrate or rhizobial density (Checcucci et al., 2016; Daubech et al., 2017; Mendoza-Suárez et al., 2020). The ability of rhizobia to form nodules can be measured during single inoculation experiments (nodulation proficiency) or in co-inoculation experiments (nodulation competitiveness). There can be significant discrepancies between these two types of assays, as the outcome of nodule occupancy following co-inoculations is unpredictable from data in single inoculation. Indeed nodulation competitiveness is a complex trait that involves a large number of bacterial genes and functions (see section 4) and that is not fully understood yet (Younginger and Friesen, 2019; Mendoza-Suárez et al., 2021). Rhizobial strains can be more competitive for nodulation if (i) they are more efficient at colonising the rhizosphere (for example by growing faster on the available nutrient sources or by producing antimicrobial compounds that inhibit the growth of sensitive competitor strains), (ii) they are faster to reach the root and induce nodulation, and/or (iii) they show an optimal compatibility with the host plant (Handelsman et al., 1984; Kiers et al., 2013; Boivin and Lepetit, 2020; Mendoza-Suárez et al., 2021). Moreover, when the co-inoculated strains have different nitrogen fixation efficiencies, the absolute and relative numbers of nodules formed by one plant can be modulated by the mechanism of auto-regulation of nodulation (AON, see section 5). Indeed, in single inoculations, strains that are fixing large amounts of nitrogen may form a relatively small number of nodules (since the nitrogen needs of the plants will be covered with few nodules), while strains that fix low amounts of nitrogen may form a large number of nodules. Yet, co-inoculating these two types of strains may modulate the number of nodules formed by each strain (Daubech et al., 2017; Westhoek et al., 2017).

Second, bacterial proliferation within nodules can be assessed by harvesting, crushing surface-sterilised nodules and plating the resulting suspensions on selective media. An important aspect of these experiments is that only viable nodule bacteria will be detected. In certain legumes, bacteroids undergo a process of 'terminal differentiation' characterised by drastic morphological and physiological changes as well as a loss of viability (Mergaert et al., 2006). As a result, the proportion of viable bacteria within nodules can vary from less than 1% in pea or *Medicago* to almost 100% in plants where bacteroids are not terminally differentiated such as soybean, with intermediate cases (Gresshoff and Rolfe, 1978; Mergaert et al., 2006; Marchetti et al., 2011). When terminal differentiation occurs, viable bacteria that can be recovered from nodules most likely arise from bacteria that were still located in infection threads or intercellular spaces at the time of harvest.

In addition, the proliferation and/or viability of rhizobia within nodule cells can be affected by their nitrogen fixation activity. Since the first discovery that soybean can ‘sanction’ non-fixing rhizobia (Kiers et al., 2003) and thereby promotes fitness of nitrogen-fixing ones, ample experimental data from several model species has accumulated to support the idea that there is a positive correlation between nitrogen fixation and rhizobial fitness (Kiers et al., 2006; Oono et al., 2011; Friesen, 2012; Kimbrel et al., 2013; Berrabah et al., 2015; Daubech et al., 2017; Quides et al., 2017; Burghardt et al., 2018; Westhoek et al., 2021; Batstone et al., 2022; Epstein et al., 2023). Plants can even discriminate fixing versus non-fixing strains in mixed nodules, and only target non-fixing bacteria for premature degeneration (Daubech et al., 2017; Regus et al., 2017). However, there are exceptions, where non-fixing rhizobia show higher fitness than fixing ones [(Crook et al., 2012; Porter and Simms, 2014; Gano‐Cohen et al., 2019) but see (Frederickson, 2020; Wendlandt et al., 2022) to ponder two of these examples]. Nodule size or weight are sometimes used as convenient proxies for bacterial fitness, but in certain cases, these values should be interpreted with caution. Indeed, the efficiency of nitrogen fixation (and the occurrence of plant ‘sanctions’, see below) or the accumulation of storage compounds can modify the relationship between nodule weight and the number of viable bacteria per nodule (Oono et al., 2011; Ratcliff et al., 2011). Another study showed, using a large collection of natural *S. meliloti* isolates, that nodule weight was positively correlated to the symbiotic fitness of the bacteria but only in one of the two plant genotypes tested (Batstone et al., 2022).

At the final stage of the symbiosis, nodules senesce and bacteria return to the soil where they can survive for months or even years before re-infecting a new host (Denison and Kiers, 2011). This extended phase of the rhizobial life cycle is crucial for the ecology and evolution of these bacteria, but remains largely understudied. In aged nodules of *M. sativa*, a population of saprophytic bacteria develop in the proximal zone (Timmers et al., 2000). A recent transcriptomic analysis showed that cell division and the general stress response are activated in rhizobia from senescent nodules (Sauviac et al., 2022). These results indicate that exiting nodules is an active process for rhizobia, but there are, to our knowledge, no study that analysed the determinants of rhizobial fitness during this phase. To persist in the soil, some bacteria may rely on previously accumulated carbon storage particles, such as polymers of poly-3-hydroxybutyrate (PHB) (Müller-Santos et al., 2021). Among rhizobial strains, some, but not all (Trainer and Charles, 2006; Chen et al., 2023), were shown to accumulate high amounts of PHB in nodules, which can represent up to 50% of the dry weight of the cells (Bergersen et al., 1995; Tavernier et al., 1997). This stored PHB can support several divisions of bacteria or a much longer survival of dormant cells without the need for any other carbon sources (Muller and Denison, 2018). Therefore, measuring PHB content in bacteroids could be a good proxy for estimating the ability of rhizobia to reproduce in soil, and an indicator of the ‘quality’ of the progenies released from nodules (Ratcliff et al., 2008; Ratcliff et al., 2011; Muller and Denison, 2018). Alternative polymers such as glycogen can also be produced by some rhizobia in nodules to store carbon and energy (Lodwig et al., 2005; Wang et al., 2007a). These processes, which consume significant amounts of energy resources, are thought to divert energy from nitrogen fixation (Lodwig and Poole, 2003) and thus induce plant sanctions (Oono et al., 2020). However, in the literature, mutants defective in the synthesis of PHB have shown contrasting nitrogen fixation phenotypes, ranging from increased to decreased nitrogen fixation (Cevallos et al., 1996; Lodwig et al., 2005; Wang et al., 2007a; Wang et al., 2007b; Crang et al., 2021). Considering the redox balance and oxygen-limiting conditions that prevail in nodules, the accumulation of PHB or other carbon polymers may indeed be required for the persistence of bacteroids in nodules (Terpolilli et al., 2016; Schulte et al., 2021). There may therefore be a trade-off between rhizobial survival in the soil, persistence in nodules and nitrogen fixation efficiency (Ratcliff et al., 2008), but these effects might be species-specific and host-plant dependent (Chen et al., 2023).

Beyond the analysis of individual fitness components, an important question relates to the potential couplings or trade-offs between the different phenotypic traits. In particular, there is no selection of nitrogen fixation at the nodulation step (since nitrogen fixation occurs at the late stages of the interaction) (Daubech et al., 2017; Westhoek et al., 2017), but several lines of evidence indicate that co-evolutionary processes may lead to the selection of strains that are both competitive for nodulation and efficient for nitrogen fixation on a given host genotype (Burghardt et al., 2018; Younginger and Friesen, 2019; Batstone et al., 2020; Fields et al., 2023; Rahman et al., 2023). This can occur through partner-fidelity feedback, a positive feedback loop acting on the fitness of the two mutualistic partners as a result of repeated associations between these organisms (Sachs et al., 2004; Fujita et al., 2014).

Although most of the fitness measurements described above usually rely on traditional microbiological techniques [with potential new optimisations, e.g. (Mendoza-Suárez et al., 2020; Quides et al., 2023)], new approaches based on bacterial populations carrying short DNA tags, in combination with next generation sequencing, now enable to analyse rhizobial fitness in a high-throughput manner (Burghardt et al., 2018; Wheatley et al., 2020). These approaches are particularly powerful to gather integrative measures of fitness (*e.g.* encompassing all fitness components composing the entire life cycle) in genetically complex rhizobial populations, and open many opportunities to perform integrative fitness measurements in a variety of conditions (Burghardt, 2020; Burghardt et al., 2020; Burghardt et al., 2022).

**Figure 1:**
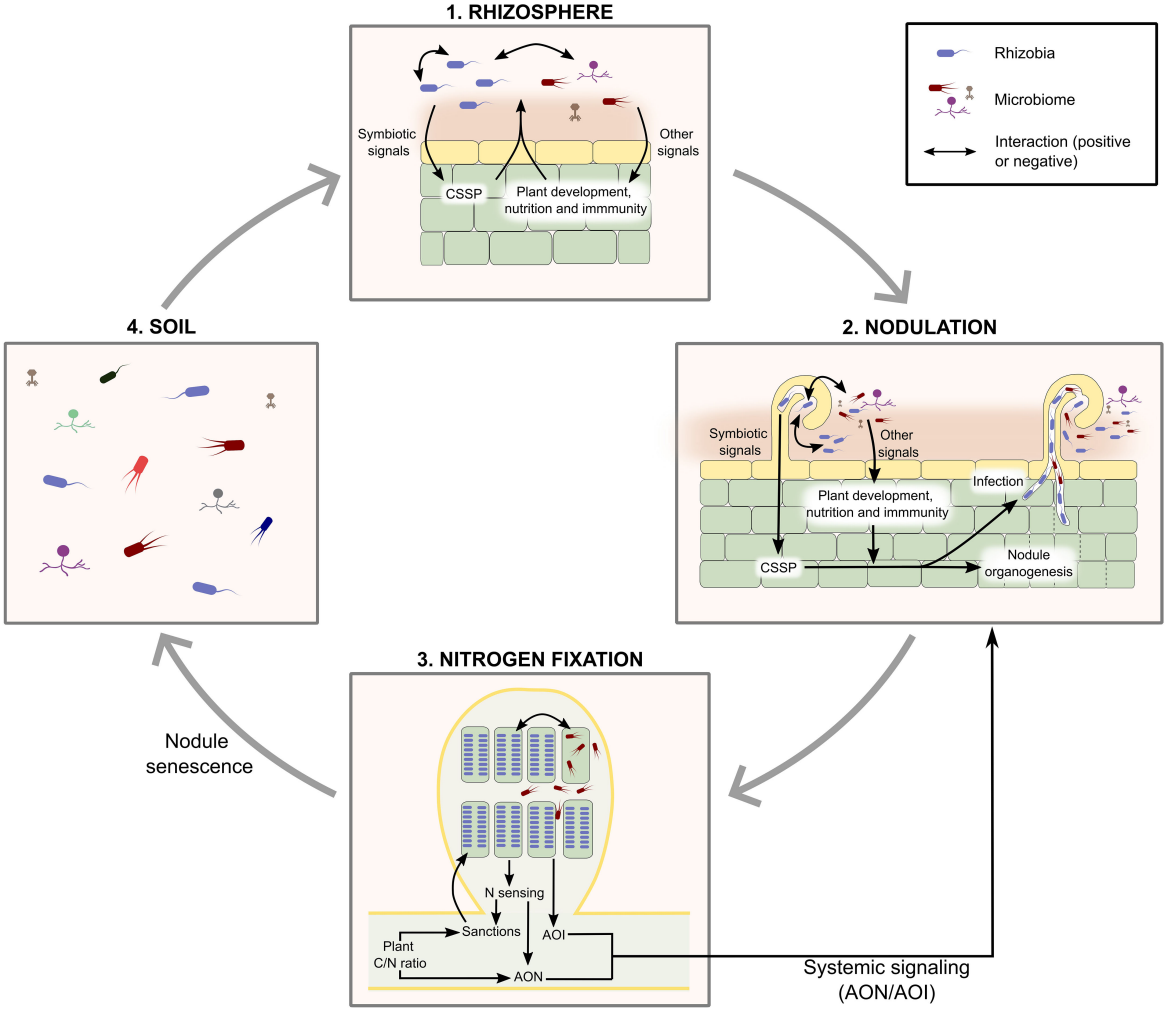
The life cycle of rhizobia and interactions with microbiome. A simplified view of the life cycle of rhizobia considers 4 successive steps: (1) colonisation of the rhizosphere, (2) initiation of nodulation, (3) nodule infection and nitrogen fixation, (4) nodule senescence and release of bacteria in the soil. Interactions with other microbes can occur at each step of the life cycle. (1) Rhizosphere: direct interactions (contact-dependent, or mediated by the secretion of beneficial or harmful chemical compounds) can occur as in any microbial community; indirectly, rhizobia and some members of the rhizosphere microbiome can modify the composition of their microbial community through plant-mediated effects: rhizobia and AMF through CSSP and non-rhizobia likely via other pathways linked to plant development, immunity, or nutrition. (2) Nodulation: rhizobia elicit plant symbiosis signaling pathways, more or less efficiently, which results in different levels of competitiveness. Other microbiome members can also induce physiological responses (for example through the secretion of plant hormones, or the modulation of hormone synthesis by the plant, or the synthesis of incompatible Nod Factors) that affect nodulation (positively or negatively, respectively). Direct physical or biochemical interactions between rhizobia and other microbes can also affect rhizobial nodulation success. (3) Nodule infection and nitrogen fixation: direct interactions can occur through the secretion of diffusible molecules (siderophores, antibiotics…) within nodules. Indirect interactions can result from the conditional sanctioning of rhizobia fixing less nitrogen than rhizobia present in other nodules. Of note, bacteria within nodules can influence the nodulation process (through AON, AOI, or the production of rhizobitoxin). AON, and probably conditional sanctioning, depend on the general physiological status of the plant (C/N ratio), which can be influenced by many biotic or abiotic factors. (4) Although we have not found any documented example (and are therefore not represented on this figure), it is also possible that in nodules indirect interactions between non-rhizobial endophytes and rhizobia occur, if nodule endophytes can activate plant signaling pathways that modify nodule physiology and rhizobial survival. Finally, although interactions likely occur during nodule senescence and bacterial release in the soil, we have not found any references on that topic. AOI, Auto-regulation of Infection; AON, Auto-regulation of Nodulation; CSSP, Common Symbiotic Signaling Pathway (induced by both AMF and rhizobium symbionts).

Finally, it is worth remembering that measurements of bacterial fitness will likely be dependent on the abiotic and biotic environment during the experiment. Abiotic factors include experimental conditions (light, temperature, plant growth substrate…), and biotic factors include host plant genotype and microbial communities (composition and density), which is the focus of this review. In the next sections, we illustrate how microbial communities affect rhizobial fitness at the different steps of their life cycle (**Figure 1**; **Table 1**; **Supplementary Table 1**).

**Table 1:** Types of microbial interactions that can alter the fitness of rhizobia^a^.

Compartment / lifecyle stage	Type of interactions	Effects/Mechanisms
Rhizosphere	Direct	A variety of microorganisms, such as rhizospheric bacteria (including Actinobacteria, nodule endophytes, or other rhizobia), yeasts, bacteriophages, protozoa or fungi, can directly interact with rhizobia in the rhizosphere, and promote or inhibit their growth. Antagonistic effects include predation, production of antimicrobial compounds, or contact-dependent inhibition, while positive interactions can occur through cross-feeding or protection by the production of biofilms.
Rhizosphere	Indirect	The composition of root exudates strongly affect microbial communities in the rhizosphere. Some rhizospheric bacteria can modify the secretion of root exudates and it is thus plausible (but, to our knowledge, remains to be shown) that these modifications can then alter the growth and survival of rhizobia in the rhizosphere.
Initiation of nodulation	Direct	Direct interactions specifically acting on the initiation of nodulation (i.e., not being a direct consequence of the rhizospheric stage) can occur through the modification of rhizobial gene expression, or the propagation of rhizobia towards infection sites thanks to fungal hyphae. Negative interactions can occur by competition for attachement sites on root hairs or the saturation of plant receptors with incompatible Nod Factors.
Initiation of nodulation	Indirect	The outcome of competition for nodulation between rhizobia will depend on the compatibility of bacterial factors (NF, LPS, EPS…) with the host plant. Cases of positive indirect interactions can occur when one rhizobial strain produces compatible diffusible signals (NF, EPS) that promote nodulation of another rhizobium (trans-complementation). Other microorganisms can interfere with nodulation by producing compounds, such as plant hormones, that favor or inhibit nodulation, or by providing nutrients that modify the global physiological status of the plant (e.g. phosphate supply by AMF).
Nodule	Direct	Nodule epiphytic bacteria assists the bacteroids in assimilating metals, likely by producing siderophores that sequester them and then transfer them to the bacteroids.
Nodule	Indirect	The persistence of rhizobia within nodules can be modified by the plant according to the nitrogen fixation level of the strains colonising the different nodules of a host plant (conditional sanctions). AMF reduces the effect of drought stress on nodule senescence by reducing oxidative stress. Rhizospheric bacteria or rhizobia can secrete molecules that interfere with plant hormonal pathways to delay nodule senescence.

a See **Supplementary Table 1** for additional details on the interactions mentionned in this table.

## 3 The rhizosphere microbiome: a hotspot for microbial interactions

The rhizosphere, which is the soil surrounding and under the influence of the roots, is characterised by a high microbial biomass and a great diversity of tens of thousands of species (Berendsen et al., 2012; De La Fuente Cantó et al., 2020). It is composed of eukaryotic microorganisms (protozoa, fungi, oomycetes, yeasts and nematodes), prokaryotic microorganisms (bacteria and archaea) and viruses (Philippot et al., 2013). This complex microbial community is highly dynamic and depends on the soil chemical and biochemical characteristics, the plant genotypes, and the type of interactions between plants and microorganisms (mutualistic, pathogenic, saprophytic, or commensal) (Philippot et al., 2013; Trivedi et al., 2020). In particular, exudates released by the roots, such as the secretion of aromatic organic acids that are preferentially consumed by rhizosphere bacteria have a major effect on microbial abundance and composition (Bais et al., 2006; Shi et al., 2011; Zhalnina et al., 2018). Abiotic factors such as pH, salinity, the presence of biofertilizers, heavy metals and carbon resource availability are also strong factors influencing the rhizobiome (Ofek et al., 2006; Bellabarba et al., 2019). In addition, microbial interactions among community members have a fundamental effect on the rhizobiome composition (Hassani et al., 2018; Chepsergon and Moleleki, 2023). Network community analyses have found co-occurrence and exclusion patterns suggesting positive and negative interactions between microorganisms (Han et al., 2020). Several mechanisms can explain these patterns. For instance, competition between organisms sharing the same ecological niche and exploiting the same resources can lead to the exclusion of some strains (Hibbing et al., 2010; Ghoul and Mitri, 2016). The secretion of antibiotics or metabolites or even predation (i.e. killing and consuming the prey) by members of the community also influence the abundance of the other members (Granato et al., 2019; Peterson et al., 2020). On the other hand, cooperation by the production of public goods (Smith and Schuster, 2019) and cross-feeding between microorganisms (D’Souza et al., 2018; Jacoby and Kopriva, 2019) can maintain the co-occurrence of some species in the community. Plant-mediated indirect interactions can also occur when microbes induce a modification in the secretion of root exudates, which in turn affects other members of the microbiome (De La Fuente Cantó et al., 2020; Korenblum et al., 2020).

The root-associated rhizobial density varies in nature (Yan et al., 2014). It depends on both positive and negative interactions with other microorganisms. Among negative interactions, predation by protozoa or bacteria (*Bdellovibrio* or *Myxococcus*) can alter rhizobial populations (Danso et al., 1975; Keya and Alexander, 1975; Ramirez and Alexander, 1980). Soil bacteriophages, which have been shown to rapidly adapt to local bacterial host communities, can also reduce the rhizobial density in the rhizosphere (Van Cauwenberghe et al., 2021). Finally, the presence of antimicrobial compounds produced by other rhizosphere microorganisms such as antibiotics produced by Actinomycetes is likely to inhibit the growth of rhizobia (Patel, 1974; Pugashetti et al., 1982). However, certain rhizobia have the means to survive to these attacks and compete with other microorganisms in the soil. In an *in vitro* experiment, Pérez et al. (2014) showed that some strains of *Sinorhizobium meliloti* that produce the exopolysaccharide (EPS) galactoglucan are more resistant to predation by *Myxococcus xanthus*. Given that EPS play a crucial role in plant recognition and early steps of symbiosis, it is possible that in the rhizosphere, EPS production has a dual ecological advantage in reducing predation and promoting interactions with compatible host plants. The production of melanin was also shown to favour resistance against predation by *Myxococcus xanthus* (Contreras-Moreno et al., 2020), and a transcriptomic analysis identified several other putative defense mechanisms, such as the production of surface polysaccharides and membrane lipids, the activation of efflux pumps or the induction of iron uptake (Soto et al., 2023).

In addition, studies have shown that type VI secretion systems (Bladergroen et al., 2003; De Campos et al., 2017) as well as the production of phages (Schwinghamer and Brockwell, 1978; Joglekar et al., 2023) or bacteriocins (Hirsch, 1979; Triplett and Barta, 1987; Oresnik et al., 1999) in rhizobia can play an important role in the direct inhibition of bacterial competitors. Among antagonistic molecules produced by rhizobia, diffusible quorum sensing molecules, in particular Acyl Homoserine Lactones (AHLs), can inhibit the growth of other rhizobial strains by activating LuxR-type regulators in the neighbouring strains and inducing genes leading to growth arrest (Schripsema et al., 1996; Wilkinson et al., 2002). The production of these antimicrobial compounds by rhizobia offers a competitive advantage in nutrient-limited environments but also for the establishment of symbiosis, by inhibiting other nodulating bacteria. A recent *in vitro* study suggests that both facilitative and inhibitory interactions exist even between rhizobial genotypes of the same species (Fields et al., 2022).

Besides negative interactions, cases of cooperation between rhizobia and other microorganisms are frequently found. An example of positive interaction was recently observed in co-cultures of the rhizobium *Rhizobium etli* and the yeast *Saccharomyces cerevisiae.* This interaction led to the formation of mixed biofilms in which the growth of the rhizobium was promoted (Andrade-Domínguez et al., 2021). This study has shown that this synergism is an effect of commensal interactions, where the rhizobia benefit from compounds secreted by the yeast such as dicarboxylic acids that bacteria use for their nutrition, and sophoroside, an antimicrobial compound detoxified by *R. etli*, which can shape the composition of the microbial community associated with this yeast. In the rhizosphere, nutritional interdependencies and reciprocal exchange of metabolites between microorganisms are commonly found (Jacoby and Kopriva, 2019). For example, a feeding of rhizobia by *Actinobacteria* capable of hydrolyzing cellulose has been evidenced (Silva et al., 2019). Other synergistic interactions were observed between *Mesorhizobia* and *Actinobacteria* (Vo et al., 2021), and between *Sinorhizobia* and bacteria of the *Bacillus cereus* group (Han et al., 2020), which enhance the growth of rhizobia by mechanisms that have not yet been elucidated.

Finally, recent research has led to the discovery that plant functional genes, and in particular symbiotic signaling genes in legumes, are involved in modulating the structure of the root-associated microbiota and the interactions among these communities (Zgadzaj et al., 2016; Thiergart et al., 2019; Liu et al., 2023). Legumes can establish mutualistic interactions with rhizobia and arbuscular mycorrizal fungi (AMF) that induce symbiotic signaling pathways involving specific components but also sharing some common components (Oldroyd, 2013). Disruption of both common and specific components of the symbiotic pathways affects the relative abundance of many bacterial and fungal taxa in the rhizosphere. For example, *Lotus japonicus* mutants defective in rhizobial NF perception (*nfr5* mutants) or in rhizobium infection (*nin* mutants) associate with a different bacterial community than the wild-type *Lotus japonicus* accession, with several bacterial orders such as Flavobacteriales, Myxococcales, Pseudomonadales, Rhizobiales, and Sphingomonadales being depleted in the roots of mutant plant genotypes (Zgadzaj et al., 2016; Wippel et al., 2021). Furthermore, inactivation of both root nodule and arbuscular mycorrhizal symbiosis pathways (*symrk* and *ccamk* mutants) was surprisingly associated with the root microbiota network structure (increase in both network connectivity and degree centrality) suggesting a higher number of interactions between community members in the absence of both symbioses (Thiergart et al., 2019). Interestingly, the activation of the specific mycorrhizal symbiosis pathway in peanut was shown to promote the accumulation of rhizobia in the rhizosphere and stimulate nodulation (Wang et al., 2021). Altogether, these studies showed a broad role of plant symbiotic genes in shaping the structure of microbial communities on legume roots or rhizosphere and the interactions between members of these communities.

## 4 Microbial interactions interfering with the initiation of nodulation

The success of nodulation by rhizobia depends not only on the rhizobial density near the root but also on a process of selection mediated by the plant. In nature, legumes are usually nodulated by a diversity of rhizobial strains that produce NF with compatible structure. However, among compatible strains, even from the same species complex, some are more competitive than others to form nodules (Elliott et al., 2009; Melkonian et al., 2014; Batstone et al., 2017; Boivin et al., 2020; Boivin et al., 2021). This preferential association is dependent on both the plant genotype, the bacterial genotypes and the interaction between the plant and bacterial genotypes (Burghardt et al., 2017; Boivin et al., 2021; Fagorzi et al., 2021). On the bacterial side, using classical genetics or large-scale transposon insertion mutant libraries, several studies have shown that hundreds of genes are involved in the competitive ability of rhizobia to form nodules (Pobigaylo et al., 2008; Wheatley et al., 2020; Mendoza-Suárez et al., 2021). Beyond NF biosynthesis and regulatory genes, genes involved in motility and chemotaxis, or other functions that help bacteria to migrate near the roots (Caetano-Anollés et al., 1988; Wheatley et al., 2020; Ji et al., 2023), and in surface polysaccharide biosynthesis (Pobigaylo et al., 2008; Janczarek et al., 2009) were shown to be important for competitive nodule formation. Also, the bacterial capacity to metabolise components found in root exudates such as the homoserine or mimosine (Soedarjo and Borthakur, 1998; Vanderlinde et al., 2014), particular sugars such as erythritol (Yost et al., 2006; Wheatley et al., 2020), rhamnose (Oresnik et al., 1998), myo-inositol (Fry et al., 2001) or amino acids such as proline (Jimenez-Zurdo, 1995) contribute to nodulation competitiveness. In addition, genes involved in purine biosynthesis (Xie et al., 2009; Wheatley et al., 2020) and nitrogen metabolism (Wheatley et al., 2020) play a role in competitive nodule occupancy. Although in nature strains that exhibit high competitiveness for nodulation often demonstrate high nitrogen fixation efficiency as well (Burghardt et al., 2018; Fields et al., 2023; Rahman et al., 2023), the capacity to fix atmospheric nitrogen itself does not contribute to nodulation competitiveness (Amarger, 1981; Hahn and Studer, 1986; Daubech et al., 2017; Westhoek et al., 2017; Bourion et al., 2018). Interestingly, co-inoculation of highly competitive and efficient nitrogen-fixing strains with another rhizobial strain can lead either to higher plant benefits than in single inoculation or, on the contrary, to a decrease in both nodulation and plant benefits, indicating the existence of positive and negative competitive interferences between the rhizobial strains (Heath and Tiffin, 2007; Montoya et al., 2023; Rahman et al., 2023). Other interactions negatively affecting nodulation initiation were described by Gano-Cohen et al. (2016), who showed that the presence of non-nodulating *Bradyrhizobium* reduced the number of nodules formed by nodulating *Bradyrhizobium* on the legume *Acmispon strigosus*, probably by directly competing for attachment sites on the root. In another study, a phenomenon called “competitive nodulation blocking” was observed in the presence of some rhizobial strains that produce high levels of NF of incompatible structure, thus inhibiting nodulation by other compatible symbionts (Hogg et al., 2002). The presence of plant pathogens can also inhibit nodule formation as shown under laboratory conditions by Benezech et al. (2021) using the *Medicago*-*Sinorhizobium*-*Ralstonia* model system.

On the other hand, direct cooperations between rhizobial strains are possible through the production of extracellular diffusible molecules that can functionally complement each other. For example, a *Mesorhizobium loti* strain deficient in NF production (R7A *nodA* mutant) and another one deficient in exopolysaccharide production (R7A *exoU* mutant) are both unable to nodulate *Lotus japonicus* but can complement each other to form functional nodules. In that case, 80% of nodules contained both strains (Kelly et al., 2013). Similar results were obtained with *nod* and *exo* mutants of *Sinorhizobium meliloti* nodulating alfalfa (Klein et al., 1988; Kapp et al., 1990). A more recent study showed that complementation of rhizobium EPS deficient strain can be done by non rhizobial nodule endophytic bacteria (Zgadzaj et al., 2015). Another example of cooperation has been observed between the *Phaseolus vulgaris* symbiont *R. etli* and a commensal rhizobium *R. fabae*. In this cooperation, *R. fabae* produces AHLs, quorum sensing molecules that are perceived by the CinR regulator of *R. etli* leading to changes in gene expression and optimisation of nodulation (Miao et al., 2018).

In addition, the presence of plant growth-promoting rhizobacteria (PGPR) in the soil can indirectly increase the nodulation efficiency of rhizobia by producing or decreasing the amounts of phytohormones known to interfere either positively or negatively with the nodulation process (Alemneh et al., 2020). For example, the production of indole-3-acetic acid (IAA), a plant hormone of the auxin class, by bacteria of the genera *Azospirillum*, *Micromonosperma*, *Pseudomonas* or *Bacillus*, increases nodule formation by rhizobia on legumes (see (Alemneh et al., 2020) for a review on bacteria able to synthesise IAA). Indeed, these molecules are known to increase lateral root growth (Casimiro et al., 2001), potentially providing more infection sites for rhizobia. IAA may also promote nodulation through induction of a rhizobium infection signaling pathway in root hairs (Breakspear et al., 2014). In addition, many PGPRs, such as bacteria of the genera *Serratia*, *Arthrobacter* or *Pseudomonas*, are able to produce 1-aminocyclopropane-1-carboxylate (ACC) deaminase which sequesters and cleaves the plant ethylene precursor ACC, reducing levels of ethylene, a negative regulator of nodule formation, and thus promoting nodulation (see (Alemneh et al., 2020) for a review on bacteria able to synthesise ACC deaminases). This process is even more important under stressful conditions, such as salinity stress, known to increase the levels of plant-produced ethylene (Ahmad et al., 2011). Finally, some PGPR can increase the availability of nutrients for rhizobia and can stimulate their growth *in vitro* (Le et al., 2016; Vo et al., 2021). An example of cooperation between *Streptomyces* and *Rhizobium* sp. was described by Tokala et al. (2002), where iron- and molybdate-sequestering siderophores produced by *Streptomyces* provide these metals to the rhizobium strain at early and late symbiotic stages, increasing both nodulation and nitrogen fixation.

Rhizobia can also interact with eukaryotic microorganisms such as fungi. A large number of studies have shown the positive effect of AMF on nodulation (Antunes and Goss, 2005; Barea et al., 2005; Pang et al., 2023; Tsikou et al., 2023). Phosphate supply by the fungus seems to be the main factor improving the rhizobium-legume symbiosis, although other factors such as the modulation of plant hormone levels by fungi, as described for PGPRs, also contribute to increase nodule number and plant biomass. Nevertheless, a meta-analysis showed that the synergistic effect between AMF and rhizobia is mostly observed in perennial plants but was not observed in annual plants (Primieri et al., 2022). These results suggest that the effect of the tripartite interaction between legumes, AMF and rhizobia can be influenced by the life history of the host plant. Additionally, these effects can vary based on the co-inoculated species, suggesting a particular compatibility between the symbionts is necessary to obtain combined positive effects in plants (Xavier and Germida, 2003), and on abiotic conditions (Afkhami et al., 2020). For example, under limited light, co-inoculation of rhizobia and AMF can lead to a reduction in plant growth, likely due to the important allocation of carbohydrates to the symbionts (Ballhorn et al., 2016). Finally, fungal mycelia form dispersion networks, which may facilitate root invasion. This was observed for a *Bradyrhizobium* strain, which initiates nodulation on peanut by a crack entry process and uses mycelia produced by a biotrophic fungus, *Phomopsis liquidambaris*, to migrate to legume rhizosphere and reach infection sites (Zhang et al., 2020).

## 5 Direct and indirect inter-bacterial interactions within nodules

Nodules represent a specific niche for rhizobia, in which they are largely shielded from competition with other soil bacteria. However, this isolation is not strict. The presence of non-rhizobial strains in nodules has been reported for a long time, but the recent advent of large-scale sequencing experiments provided some more complete and systematic descriptions of these 'nodule-associated bacteria' (NAB). These findings open the possibility that rhizobia may interact directly with other non-rhizobia within nodules.

Reported NAB belong to many different genera of Alpha-, Gamma- or Beta- proteobacteria (the latter now been included as a subset of Gamma-proteobacteria (Parks et al., 2018)) as well as to the Actinobacteria and Firmicutes phyla (Martínez-Hidalgo and Hirsch, 2017). NAB tend to have lower densities than rhizobia within nodules (Etesami, 2022), but their precise localisation within nodules is often unknown. Numerous studies have shown that NAB can affect the number of nodules formed by rhizobia and/or plant growth. In fact, many NAB seem to possess plant-growth promoting traits, making them good candidate as inoculants together with selected rhizobial strains (Martínez-Hidalgo and Hirsch, 2017; Velázquez et al., 2017; da Silva et al., 2023). Yet, there is little information currently available on the activity and biological relevance of these NAB within nodules.

In *Lotus*, NAB can enter nodules by co-colonising infection threads formed by the compatible rhizobium *Mesorhizobium loti* (Zgadzaj et al., 2015). A fluorescently labeled non-nodulating and non-fixing strain *Rhizobium mesosinicum* KAW12 was then shown to colonise both inter- and intra-cellular nodule spaces, when co-inoculated with *M. loti*. Colonisation was dependent on the symbiotic genetic program of the host plant, activated by the compatible NF from *M. loti.* Crosbie et al. (2022) later identified an antagonistic interaction between another intracellular nodule commensal (*Pseudomonas* sp.) and an ineffective *Rhizobium* strain. Indeed, *Pseudomonas* strains were detected in *L. japonicus* nodules formed by an efficient *M. loti* strain, but not in nodules formed by the non-fixing *Rhizobium* sp. BW8-2. Co-inoculating one of these *Pseudomonas* strains with *Rhizobium* sp. BW8-2 reduced the number of nodules formed by the latter strain, suggesting a negative effect of *Pseudomonas* on *Rhizobium* during the early stages of symbiosis. This effect was observed on *Lotus japonicus* but not on *L. burnetii*, showing that the host plant plays a role in the mediation of this rhizobium-NAB interaction.

Hansen et al. (2020) were also able to isolate cultivable NAB from *Medicago sativa* nodules formed by *S. meliloti.* These strains, representative of a simplified nodule microbial community established after three cycles of *in planta* selection, were used to perform functional studies on synthetic communities, by measuring the rate of nodule colonisation of each strain when co-inoculated with other members of this community (but always including *S. meliloti*). Both cooperative and antagonistic interactions between strains were identified in this system. Several interesting results emerge from this study. First, while two strains (*Pseudomonas* sp. and *Paenibacillus* sp.) showed a mutually beneficial effect on the rate of nodule colonisation, *Paenibacillus* sp. proliferation within nodules was reduced in the presence of *Pseudomonas* sp., showing that the type of interaction can be dependent on the phase of the life cycle. Second, all endophytes were able to antagonise *S. meliloti* in *in vitro* conditions and reduced nodule formation. However, co-inoculation of the entire community with *S. meliloti* reduced neither nodule formation nor plant growth, suggesting that higher-order interactions modify the effect of these NAB on *S. meliloti*. Finally, spatial metabolic analyses indicate that some antagonistic interactions might be mediated by the production of antibiotics by one of the nodule commensal strains.

Overall, these three studies gathered compelling evidence that rhizobia-NAB and rhizobia-plant-NAB interactions do occur within nodules. Future investigations on the precise effects of NAB on rhizobial fitness and nitrogen fixation will undoubtedly complete our understanding of these fascinating aspects of rhizobia biology.

Direct interactions between rhizobia and other bacteria may also occur at longer distance. A subset of rhizobial species produce rhizopines, a class of inositol-derived molecules synthesised in bacteroids (Murphy et al., 1995). Rhizopines are believed to be catabolised by rhizobia that are in the rhizosphere or infection threads and carry the corresponding degradation operons. In agreement with this hypothesis, rhizopine-catabolising strains have a fitness advantage over non-catabolizing strains during symbiosis (Gordon et al., 1996). However, this advantage was observed at early symbiotic stages, and it is therefore unclear if it arises from the ability to catabolise rhizopines produced by bacteroids, or other compounds produced by plants or bacteria in the rhizosphere. Nevertheless, the ability to catabolise rhizopines was also found in non-rhizobia (Gardener and De Bruijn, 1998), suggesting that other rhizospheric bacteria or possibly NAB may benefit from rhizopine produced by nodule bacteria and that rhizopines may represent a ‘public good’ for rhizospheric bacteria (although the capacity to catabolise it is restricted to a relatively small subset of bacteria). This consideration opens interesting evolutionary questions that have been addressed by mathematical modeling (Simms and Bever, 1998).

Indirect long-distance interactions also occur between rhizobia present within nodules and rhizobia located in the rhizosphere. The process of auto-regulation of infection (AOI) initiated by nodule rhizobia regulates root-hair infection by rhizospheric bacteria (Tian et al., 2012). This phenomenon involves complex signal exchanges between plants and nodule bacteria (Garnerone et al., 2018; Sorroche et al., 2019; Sorroche et al., 2020). However, the relevance of AOI for rhizobial fitness when plants are exposed to different rhizobial or non-rhizobial strains remains to be investigated. In addition, infection and nitrogen fixation by rhizobia have strong effects on plant physiology, and can lead to the production of systemic signals that modulate rhizobial fitness throughout the plant (Lepetit and Brouquisse, 2023). For example, the auto-regulation of nodulation (AON) allows the plant to block the nodulation process when its needs in nitrogen are covered by the functioning nodules (Ferguson et al., 2019). Long-distance interactions are also known to occur between rhizospheric microorganisms and nodule bacteria (Ruiz-Lozano et al., 2001; Tokala et al., 2002). In soybean, symbiosis with the AMF *Glomus mosseae* protects nodules from drought-induced senescence (Ruiz-Lozano et al., 2001). The authors observed that AMF reduced the effect of drought on several markers associated with nodule senescence, such as the decreased nitrogen fixation activity and the early appearance of oxidative damages. It is likely that this protective effect also translates into better rhizobial fitness.

Finally, indirect interactions between rhizobia present in different nodules have recently been reported when plants are colonised by several different rhizobial strains. While it is now well established that rhizobial persistence within nodules depends on their rate of nitrogen fixation (see section 2), Whesthoek et al. (2021) showed that this effect can be modulated depending on the symbiotic effectiveness of the diverse strains nodulating the same host plant. Using a strain that fixes reduced amounts of nitrogen (‘intermediate fixer’), the authors showed that bacterial load within mature nodules colonised by this strain is lower when the plant is co-inoculated with an efficient strain than with a non-fixing strain. These results show that plants are able to impose 'conditional sanctions' by comparing nitrogen output from the different nodules and responding accordingly in ways that affect rhizobial survival within nodules. This response seems to involve differential transport of sugars and di-carboxylic acids to the nodules, but the precise molecular mechanisms involved in the sensing of differential nitrogen fixation levels and the establishment of conditional sanctions remain to be discovered.

## 6 Towards the incorporation of ecological interactions into evolutionary processes

All the examples mentioned so far in this review (and also reviewed by others; (Barea et al., 2005; Afkhami et al., 2020; Checcucci and Marchetti, 2020; Burghardt and diCenzo, 2023)) show that rhizobial fitness can be modified by the presence of other microorganisms at the different steps of the life cycle. Therefore, microbial communities will change the selective pressures acting on rhizobial populations, and thus their evolutionary trajectories. Below we discuss two processes by which the biotic environment can affect rhizobial evolution: eco-evolutionary feedbacks and the alteration of the mechanisms of evolution. For more general perspectives on the evolution of rhizobia, we refer interested readers to the following recent reviews (Andrews et al., 2018; Burghardt, 2020; Denison and Muller, 2022; Remigi et al., 2016; Masson-Boivin and Sachs, 2018; Tian and Young, 2019; Tang and Capela, 2020; Provorov et al., 2022; Wardell et al., 2022; Liu S, et al., 2023).

Eco-evolutionary feedbacks refer to the interplay between the modification of selective pressures by ecological mechanisms (*e.g.* biotic interactions) and the modification of ecological interactions by evolutionary mechanisms (Schoener, 2011; Hendry, 2017; Ware et al., 2019). Eco-evolutionary feedbacks are expected to be particularly relevant when the time scales of ecological and evolutionary processes overlap, which is typically the case in microbial populations (Fields and Friman, 2022). In the case of rhizobia, the presence of a complex microbiome can shape not only the evolution of the interactions between rhizobia and other microbiome members, but also interactions between rhizobia and their host plant. This seems be the case for example with the emergence of phage-resistant rhizobia that survive better in the rhizosphere but show a decreased symbiotic performances on their host plant (Kleczkowska, 1950; Stacey et al., 1982; Handelsman et al., 1984; Mishra et al., 2004; Anand et al., 2012). In another example of eco-evolutionary feedback, the co-culture of *R. etli* with yeasts leads to the rapid appearance of bacterial phenotypic variants (Andrade-Domínguez et al., 2014). These variants carry diverse mutations that allow *R. etli* to grow in the presence of orotic acid, a metabolite secreted by yeasts and that inhibits the growth of wild-type bacteria. These variants then further evolved to become yeast antagonists, thus completely changing the nature of the interaction between these organisms. Although it is unknown whether these two strains interact with one another in their natural environment, this work nicely unravels how the presence of yeasts can rapidly affect the phenotypic and genetic composition of rhizobial populations.

Experimental evolution can also be used to study how plant-rhizobia interactions can change in a variety of ecological contexts (Remigi et al., 2019). Doin De Moura et al. (2023) analysed an evolution experiment designed to turn a plant pathogenic bacterium into a legume symbiont, following the artificial transfer of a symbiotic plasmid into the plant pathogenic ancestor (Doin de Moura et al., 2020). In the course of this experiment, multiple sub-populations of bacteria emerge and compete in the rhizosphere. Evolved bacteria (that are still not fixing nitrogen at the end of the experiment) predominantly improved their nodulation competitiveness. These results show that, in emerging rhizobia with poor symbiotic phenotypes, the selective bottleneck at the nodulation step exerts a strong selective pressure on bacterial populations.

Batstone et al. (2020) performed serial nodulation cycles on five lines of *M. truncatula* using a mix of two rhizobial strains as starting inoculum: one effective and one ineffective nitrogen-fixing strain. During this experiment, the effective strain increased in frequency in almost all lines, and became even more beneficial to the plant lines on which they evolved. An interesting follow-up of this experiment would be to evolve strains alone or in co-inoculation, to assess the effect of competition between multiple rhizobial strains on their respective evolutionary trajectories.

The presence of a complex microbial community can also modify the basic evolutionary mechanisms acting on a focal species, such as the rates of mutations and of horizontal gene transfer (HGT). Bacterial mutation rates are plastic (Ferenci, 2019; Matic, 2019; Pribis et al., 2022), and can be influenced by various stresses, nutrient availability (Maharjan and Ferenci, 2017), or cell density (Krašovec et al., 2017). Interestingly, in *E. coli*, the modulation of mutation rate by cell density depends on the quorum-sensing gene *luxS*, indicating a ‘social-dependence’ of mutation rate in this strain (Krašovec et al., 2014). Given that microbial communities can constitute stressful environments for bacteria (Hibbing et al., 2010; LeRoux et al., 2015; Traxler and Kolter, 2015; Granato et al., 2019), it is plausible that mutation rates of rhizobia might be affected by other microorganisms. In addition, many symbiotic plasmids also carry stress-inducible error-prone DNA polymerases (Remigi et al., 2014). After transferring one of these plasmids (from the rhizobium *Cupriavidus taiwanensis*) into a non-symbiotic bacterium, the expression of error-prone DNA polymerases in stressful environments (the rhizosphere) induces transient bursts in mutation rate and accelerates adaptation to the symbiotic lifestyle. Antagonistic interactions between microbiome members can also lead to the selection of constitutive hypermutator strains (*e.g.*, that are defective in DNA repair systems). This has been documented for phage-bacteria interactions where lytic phages impose strong mortality in bacterial populations. Second-order selection of constitutive hypermutators can thus occur, as these strains will have a higher chance to become resistant to phages (Chevallereau et al., 2022).

HGT is another major driver of rhizobia evolution. Evidently, dense and diverse microbial communities, like those found in the rhizosphere, facilitate HGT due to both the elevated transfer rates and the extensive pool of genes that could potentially be transferred (Brito, 2021; Moura De Sousa et al., 2023; Sánchez-Salazar et al., 2023). Yet additional mechanisms exist that further increase HGT in rhizobia. The transfer rate of symbiotic integrative and conjugative elements (ICE) from *Azorhizobium caulinodans* to other rhizobia is elevated in the rhizosphere of several plants compared to empty soil (Ling et al., 2016). The perception of flavonoid compounds by a transcription regulator of the LysR family controls the expression of the ICE integrase, together with two other genes located on this symbiotic island (Ling et al., 2016; Li et al., 2022). Recently, the transfer of symbiotic plasmids from *R. etli* CFN42 to other rhizobia and NAB was also detected in bean nodules (Bañuelos‐Vazquez et al., 2019; Bañuelos-Vazquez et al., 2020). These examples show that symbiosis genes are particularly prone to be transferred in the presence of host plants. Indeed, HGT of symbiosis genes has been extensively documented by phylogenetic studies (Remigi et al., 2016; Andrews et al., 2018; Wardell et al., 2022). In the field, non-symbiotic strains that are well adapted to the abiotic environment can become efficient symbionts by acquiring symbiotic ICE from ‘elite’ rhizobial inoculants that are not adapted to the soil conditions (Sullivan et al., 1995; Nandasena et al., 2007; Hill et al., 2021; Colombi et al., 2023). Moreover, the frequent re-arrangements and recombinations between symbiotic genes from different strains create a dynamic equilibrium where less cooperative strains regularly appear as well as strains with new or improved symbiotic abilities, illustrating how genetic diversity of rhizobial strains fuels the evolvability of symbiosis (Weisberg et al., 2022). Altogether, these studies demonstrate that incorporating the whole microbial communities will be crucial to understand the rate and patterns of rhizobial evolution.

## 7 Conclusion and perspectives

The rhizobium-legume symbiosis has long been a model system to study both the molecular and eco-evolutionary aspects of host-microbe interactions. In the recent years, we have witnessed an increased interest in incorporating the effect of complex microbial communities on the functioning of this symbiosis, and numerous interactions with a multitude of microbes have already been detected throughout the rhizobial life cycle in both laboratory and field conditions (**Table 1**).

However, certain stages of the rhizobial life cycle have been understudied. For instance, interactions within the nodule microbiome, although much less complex than the rhizosphere microbiome, still lack a comprehensive understanding. Similarly, interactions of bacteria released from senescent nodules with soil communities remain largely unknown. In addition, research has predominantly focused on pairwise interactions so far, while in nature, interspecies interactions occur in multispecies communities. In such complex ecosystems, higher-order interactions can profoundly impact the fitness of a focal strain (Levine et al., 2017). Another set of open questions relates to the genetic bases of inter-microbial interactions. Once we start to uncover the molecular mechanisms involved in these interactions and to describe the existing natural genetic variations in the life history traits, it will be particularly interesting to further test whether there are genetic trade-offs or couplings between these different traits, including those involved in the interaction with the host plant. In parallel, assessing the eco-evolutionary dynamics of these interactions with laboratory evolution or field experiments will provide complementary information on the selective pressures acting on rhizobial populations.

Therefore, we have probably only scratched the surface of the extent to which rhizobial-microbial interactions contribute to the establishment and functioning of legume-rhizobia symbioses. Additional discoveries are expected as research progresses on this topic, concerning both the mechanisms that mediate these interactions and their ecological and evolutionary consequences. A future challenge will be to integrate all these findings in a coherent framework, and use this knowledge to improve agricultural and ecosystem services that can be obtained from rhizobia. An ambitious goal would be to be able to design synthetic communities where microbial interactions enhance rhizobial fitness and symbiotic services, and mitigate the effects of plant pathogens. This will require cooperation between several disciplines (molecular microbiology, ecology and evolutionary biology, plant physiology, agronomy…) and cross-fertilisation between laboratory and field experiments.

## Author contributions

MGA: Writing – original draft, Writing – review & editing. BR: Writing – original draft, Writing – review & editing. DC: Writing – original draft, Writing – review & editing. PR: Writing – original draft, Writing – review & editing.
